# Metabolism modifications and apoptosis induction after Cellfood™ administration to leukemia cell lines

**DOI:** 10.1186/1756-9966-32-63

**Published:** 2013-09-09

**Authors:** Simona Catalani, Valentina Carbonaro, Francesco Palma, Marselina Arshakyan, Rossella Galati, Barbara Nuvoli, Serafina Battistelli, Franco Canestrari, Serena Benedetti

**Affiliations:** 1 Department of Biomolecular Sciences, Section of Clinical Biochemistry and Cellular Biology, University of Urbino “Carlo Bo”, Via Ubaldini 7, 61029 Urbino, PU, Italy; 2 Department of Biomolecular Sciences, Section of Biochemistry and Molecular Biology, University of Urbino “Carlo Bo”, Via Saffi 2, 61029 Urbino, PU, Italy; 3 Molecular Medicine Area, “Regina Elena” National Cancer Institute, Via Elio Chianesi 53, 00144 Rome, Italy

**Keywords:** Apoptosis, Cellfood™, Glucose transporter 1, Hypoxia inducible factor 1 alpha, Lactate dehydrogenase, Leukemic cells, Tumor metabolism

## Abstract

**Background:**

Cellfood™ (CF) is a nutritional supplement containing deuterium sulphate, minerals, amino acids, and enzymes, with well documented antioxidant properties. Its organic and inorganic components are extracted from the red algae *Lithothamnion calcareum*, whose mineral extract has shown growth-inhibitory effect both on *in vitro* and *in vivo* models. The purpose of this study was to evaluate the antiproliferative effects of CF on leukemic cells. In fact, according to its capacity to modulate O_2_ availability and to improve mitochondrial respiratory metabolism, we wondered if CF could affect cancer cell metabolism making cells susceptible to apoptosis.

**Methods:**

Three leukemic cell lines, Jurkat, U937, and K562, were treated with CF 5 μl/ml up to 72 hours. Cell viability, apoptosis (i.e. caspase-3 activity and DNA fragmentation), hypoxia inducible factor 1 alpha (HIF-1α) concentration, glucose transporter 1 (GLUT-1) expression, lactate dehydrogenase (LDH) activity and lactate release in the culture medium were detected and compared with untreated cells.

**Results:**

CF significantly inhibited leukemic cell viability by promoting cell apoptosis, as revealed by caspase-3 activation and DNA laddering. In particular, CF treated cells showed lower HIF-1α levels and lower GLUT-1 expression as compared to untreated cells. At the same time, CF was able to reduce LDH activity and, consequently, the amount of lactate released in the extracellular environment.

**Conclusions:**

We supplied evidence for an antiproliferative effect of CF on leukemia cell lines by inducing cell death through an apoptotic mechanism and by altering cancer cell metabolism through HIF-1α and GLUT-1 regulation. Thanks to its antioxidative and proapoptotic properties, CF might be a good candidate for cancer prevention.

## Background

Cell proliferation, that represents the essence of cancer disease, involves not only a deregulated control of cell cycle but also adjustments of energy metabolism in order to fuel cell growth and division. In fact, proliferation of cancer cells is accompanied by glycolysis activation and this altered glucose metabolism is one of the most common hallmark of cancer [1,2]. Approximately 60 to 90% of cancers display a metabolic profile, the so-called Warburg phenotype, characterized by their dependence upon glycolysis as the major source of energy, irrespective of the oxygen level [3]. According to the Warburg effect, cancer cells up-regulate glucose transporters, notably GLUT-1, and convert pyruvate, the end-product of glycolysis, into lactate by lactate dehydrogenase (LDH), rather than oxidizing it in mitochondria [4-6].

In this context, the hypoxia inducible factor 1 (HIF-1) has been shown to play a fundamental role [7,8]. HIF-1 is a transcription factor that consists of an O_2_-regulated HIF-1α and a constitutively expressed HIF-1β subunit. In cancer cells, HIF-1α is up-regulated and, in turn, activates the expression of glycolytic enzymes (such as LDH) and glucose transporters (such as GLUT-1), and down-regulates the mitochondrial activity through several mechanisms, in particular by inhibiting the conversion of pyruvate to acetyl-CoA via the activation of the gene encoding pyruvate dehydrogenase kinase 1 [7-10]*.*

Shifting metabolism away from mitochondria (glucose oxidation) and towards the cytoplasm (glycolysis) might suppress apoptosis, a form of cell death that is dependent on mitochondrial energy production [11,12]. Accordingly, the glycolytic phenotype has been associated to apoptosis resistance and consequently increased tumor cell proliferation [3,4,13].

Understanding the metabolic basis of cancer has the potential to provide the foundation for the development of novel approaches targeting tumor metabolism [14]. In this regard, recent observations suggest that the reversion of the glycolytic phenotype may render tumor cells susceptible to apoptosis and decrease their growth rate [15-17].

With this in mind, we planned to investigate whether the natural supplement Cellfood™ (CF; Nu Science Corporation, CA, USA) might have antiproliferative effects in vitro, limiting cell proliferation and promoting cell death. CF is a proprietary formulation containing 78 ionic/colloidal trace elements and minerals combined with 34 enzymes and 17 amino acids, all suspended in a solution of deuterium sulphate [18]. The organic and inorganic components of the supplement are extracted from the marine red algae *Lithothamnion calcareum*, whose mineral extract has shown growth-inhibitory effects on human colon carcinoma cells [19] as well as inhibition of liver tumor formation in C57BL6 mice [20].

Referring to CF formulation, previous studies have demonstrated its ability to furnish effective in vitro antioxidant protection [21]. At the same time, the capability of CF to modulate O_2_ availability and mitochondrial respiratory metabolism has been evidenced in endothelial cells [22].

All these observations led us to investigate the potential role of CF as hypoproliferative agent *in vitro.* For this purpose, we analyzed the effect of CF on cell growth, viability, glycolytic profile, and apoptosis on three human leukemia cell lines, Jurkat, U937, and K562. Eighteen percent of malignancies are of hematological origin [17]; moreover, leukemic cells are highly glycolytic [23], though these cells reside within the bloodstream at higher oxygen tensions than cells in most normal tissue. In the present study we reported evidence that CF showed antiproliferative effect on the above mentioned leukemia cell lines due to apoptosis induction and tumor metabolism modifications.

## Methods

### Cellfood™

The supplement (liquid) was kindly provided by Eurodream srl (La Spezia, Italy) and stored at room temperature. CF was diluted in phosphate buffered saline (PBS) and sterilized using a 0.45 μm syringe-filter before use.

### Cell culture

Three human leukemia cell lines were used in this study, Jurkat (acute lymphoblastic leukemia), U937 (acute myeloid leukemia), and K562 (chronic myeloid leukemia in blast crisis). Cells were grown in RPMI 1640 medium supplemented with 10% heat-inactivated fetal bovine serum, 1% L-glutamine and 1% penicillin/streptomycin 100 U/ml, and incubated in a CO_2_ incubator (37°C, 5% CO_2_ and humidified atmosphere). Cell culture reagents were from VWR International (Milan, Italy).

Lymphocytes were isolated from blood samples provided by healthy volunteers by centrifugation in the presence of Lymphoprep™ (Axis-Shield PoC AS, Oslo, Norway), and were cultured as described above with the addition of 10 μg/ml of phytohemagglutinin (Sigma-Aldrich, Milan, Italy).

A single dose of CF (final concentration 5 μl/ml) was administered to leukemia cells or lymphocytes; cells were collected after 24, 48, and 72 h of CF administration. Untreated cells served as controls. Trypan blue cell counting was performed at each experimental time point to evaluate the viable cell number.

### Cell viability assay

Cell proliferation and viability were analyzed at 450 nm by the WST-1 reagent (4-[3-(4-lodophenyl)-2-(4-nitrophenyl)-2H-5-tetrazolio]-1,3-benzene disulfonate) (Roche Diagnostics GmbH, Mannheim, Germany). The assay was based on the cleavage of the tetrazolium salt WST-1 by mitochondrial dehydrogenases in viable cells. Briefly, leukemia cells were incubated in 96-well plates in the presence or absence of CF (5 μl/ml); after 24, 48, and 72 h of incubation, WST-1 was added to each well, and cells were further incubated at 37°C up to 2 h. Colour development was monitored at 450 nm in a multiwell plate reader (Thermo Fisher Scientific, Shangai).

### Caspase-3 activity evaluation

Caspase-3 activity was determined in leukemia cells using a colorimetric kit from Biovision (Milpitas, CA, USA) in accordance with the manufacturer’s instructions. The assay is based on the spectrophotometric detection at 405 nm of the chromophore p-nitroaniline (pNA) after cleavage from the labeled substrate DEVD-pNA by caspase-3. Protein concentration in the cytosolic extracts was measured using the Bradford method [24].

### DNA fragmentation analysis

The genomic DNA fragmentation was evaluated by agarose gel electrophoresis of DNA isolates obtained by the salting-out method [25]. For this purpose, leukemia cells were grown in the presence or absence of CF 5 μl/ml up to 72 h; a positive control (cells treated for 6 h with 25 μM etoposide) was also included. After counting and washing, cells were subjected to DNA extraction. The DNA samples were carefully resuspended in TE buffer; the nucleic acid concentration and purity were measured using a NanoDrop® ND-1000 spectrophotometer (Thermo-Scientific, Wilminton, DE, USA). 2 μg of each sample was loaded onto 1.5% TAE agarose gel; DNA laddering was visualized on a UV transilluminator by ethidium bromide staining. Images were obtained using a Gel Doc 2000 (Bio-Rad Laboratories S.r.l, Segrate, MI, Italy).

### HIF-1α measurement

HIF-1α quantification was performed in leukemia cells using an enzyme-linked immunosorbent assay kit from Abcam (Cambridge, UK), in accordance with the manufacturer’s instructions. Colour development was evaluated at 450 nm in a multiwell plate reader (Thermo Fisher Scientific, Shangai). Protein concentration in cell extracts was measured using the Bradford method [24].

### Western blot assay of GLUT-1

Leukemia cells were grown in presence or absence of CF 5 μl/ml up to 72 h. After counting and washing, cells were resuspended in 1X SDS loading buffer to 20x10^6^ cells/ml. Cell lysis was achieved by vortex, and the viscosity was reduced by passing through a syringe needle. 15 μl of each samples were run on 0.8% SDS-polyacrylamide gel and the resolved proteins were electrophoretically transferred to supported nitrocellulose membranes (Bio-Rad Laboratories S.r.l, Segrate, MI, Italy) using a Bio-Rad Semidry Transfer system. Non-specific binding to membranes was blocked by incubation in blocking solution (50 mM Tris–HCl, 150 mM NaCl and 5% (w/v) non-fat dried milk, pH 7.5). After blocking solution removal, membranes were incubated in a new blocking solution with a rabbit polyclonal GLUT-1 antibody (PA1-46152, Thermo Scientific) at 4°C overnight. Membranes were then washed three times with TTBS (50 mM Tris–HCl, 150 mM NaCl and 0.05% (v/v) Tween 20, pH 7.5) and incubated with horseradish peroxidase-conjugated anti-rabbit secondary antibody diluted 1:4500 in TTBS for 1 h at room temperature. After TTBS washes, the blot was incubated in detection reagent (ECL Advance Western Blotting Detection Kit) and exposed to a Hyperfilm ECL film (Pierce).

### LDH activity and lactate release measurement

After 72 h of incubation in the presence or absence of CF (5 μl/ml), leukemia cells were centrifuged at 450 g for 10 min at room temperature; supernatants were collected to evaluate lactate release in the culture media while cell pellets were used for LDH activity determination.

Lactate measurement was performed through an enzymatic assay in a hydrazine/glycine buffer (pH 9.2), containing 2 mg/ml β-NAD^+^ and 16 units/ml LDH [26]. The absorbance due to NADH formation was monitored spectrophotometrically at 340 nm and the amount of lactate released in the media was calculated using the molar extinction coefficient of NADH.

To test LDH activity, cell pellets were washed once with PBS by centrifugation at 450 g for 10 min at 4°C. Supernatants were discarded and pellets resuspended in a lysis buffer (CellLytic M reagent, Sigma-Aldrich, Milan, Italy) containing a specific protease inhibitor cocktail (Sigma-Aldrich, Milan, Italy). After 15 min incubation, lysed cells were centrifuged at 12,000 g for 15 min at 4°C. The protein-containing supernatants were used for LDH activity measurement as previously described [27]. The assay medium contained 50 mM Tris–HCl, pH 8, 0,2 mM β-NADH, and 5 mM pyruvate. The oxidation of NADH was monitored as a decrease in 340 nm absorbance at 37°C. Protein concentration in cell lysates was measured using the Bradford method [24].

### Statistical analysis

The data are presented as the mean ± standard deviation of at least three experiments and analyzed using Student’s *t*-test. Significance level was set at p < 0.05 for all analysis.

## Results and discussion

Over the last decades, many studies using animal models have shown numerous dietary constituents and nutraceuticals as cancer chemopreventive agents [28]*;* in fact, it has been generally accepted that they can suppress transformation, hyperproliferation, invasion, angiogenesis and metastasis of various tumors [29]. Because oxidative and inflammatory stress contributes to malignant transformation, dietary agents with antioxidative, anti-inflammatory and proapoptotic properties would be good candidates for preventing human malignancies [30-33].

**Figure 1:**
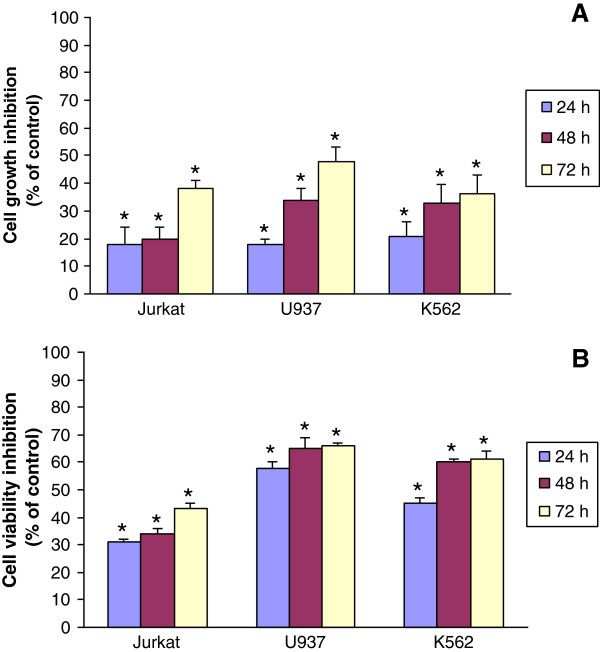
**Significant inhibition of leukemia cell proliferation (A) and viability (B) after 24, 48, and 72 h of incubation with CF in comparison with untreated cells (control), as evaluated by cell counting by trypan blue dye exclusion and WST-1 reagent, respectively.** Data are expressed as mean ± SD of at least three independent experiments. *p < 0.05 vs. untreated cells.

**Figure 2:**
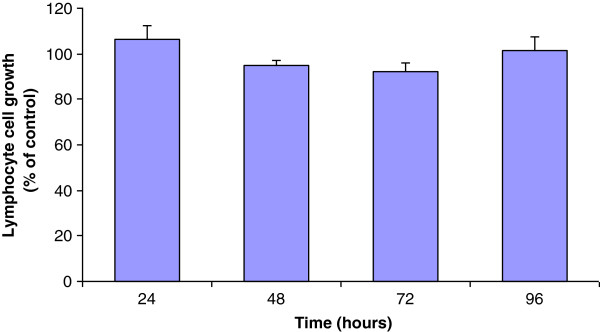
**Lymphocyte cell growth in the presence of CF (5 μl/ml) in comparison with untreated cells (control).** No effects were observed up to 96 h after CF administration to isolated lymphocytes as a non-tumor cell system Data are expressed as mean ± SD of at least three independent experiments.

Cellfood™ is a nutritional supplement whose antioxidant properties have been well documented *in vitro*[21]. In the present study, we demonstrated for the first time that in leukemia cell lines (Jurkat, U937, and K562) CF treatment reduced cancer cell proliferation and viability without affecting healthy lymphocyte growth. In fact, CF administration at the concentration of 5 μl/ml induced a significant reduction of leukemia cell growth as revealed by the vital dye trypan blue (Figure 1A). The decrease of cancer cell growth was maximum after 72 h, reaching up to 50% inhibition in U937 cell line. Cell viability was also evaluated through the measurement of mitochondrial dehydrogenase activity using the colorimetric WST-1 assay (Figure 1B). Data confirmed that CF treatment induced cell viability inhibition up-and-over 60% in U937 cells after 72 h of incubation. To investigate the selectivity of CF treatment towards tumor cells, human healthy lymphocytes were seeded in the presence of the same concentration of CF up to 96 h; data revealed no significant differences between untreated and treated cells, confirming that CF did not affect healthy lymphocyte growth (Figure 2).

These results are in accordance with the growth-inhibitory properties of *Lithothamnion calcareum*, the red algae from which the organic and inorganic components of CF are extracted [19,20]. Indeed, the mineral-rich material derived from the algae has been shown to suppress the growth of a series of human colon cancer cell lines *in vitro*[19], as well as to protect mice against neoplastic and preneoplastic proliferative liver lesions [20].

**Figure 3:**
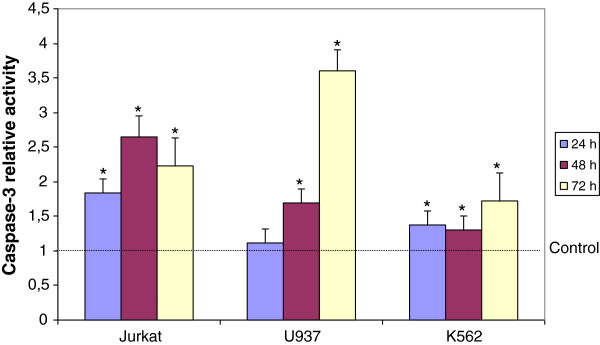
**Significant increment of caspase-3 activity in leukemia cells after 24, 48, and 72 h of incubation with CF (5 μl/ml) in comparison with untreated cells (control).** Data are expressed as mean ± SD of at least three independent experiments. *p < 0.05 vs. untreated cells.

To clarify whether CF was able to reduce cancer cell viability by promoting apoptotic cell death, two classical markers of apoptosis were determined. Caspase-3 is considered to be the most important effector of apoptosis and a marker for both intrinsic and extrinsic pathways [11]. Noteworthy, we evidenced that CF treatment significantly stimulated caspase-3 activity in the three leukemia cell lines as compared to the respective untreated controls (Figure 3).

**Figure 4:**
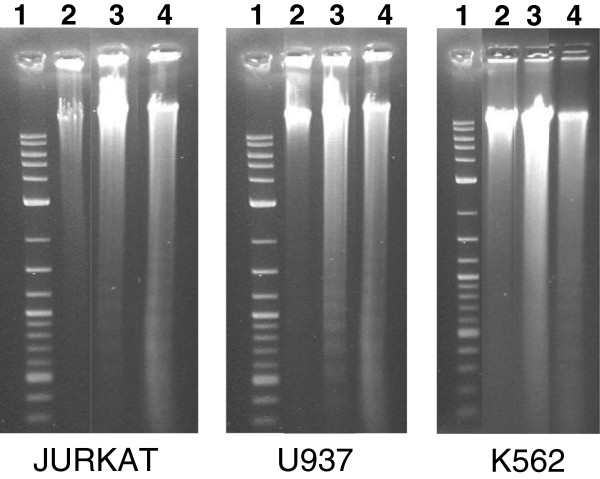
**DNA fragmentation of leukemia cells after 72 h of incubation with CF (5 μl/ml).** Apoptotic DNA fragmentation was qualitatively analyzed by agarose gel electrophoresis. Lane 1: 1 kb DNA ladder marker; lane 2: negative control (untreated cells); lane 3: CF treated cells; lane 4: positive control (etoposide).

On the other hand, the detection of the internucleosomal DNA cleavage (or DNA laddering) is a common hallmark of cells undergoing late-stage apoptosis [11]*.* To verify if CF could induce DNA fragmentation and thus to confirm whether apoptosis occurred, leukemia cells exposed to CF treatment were assessed for DNA laddering by agarose gel electrophoresis (Figure 4). We found that the three cell lines incubated with CF showed apoptotic DNA fragmentation profiles similar to the positive control, which was represented by cells incubated with etoposide that is commonly known to be an apoptosis inducer [34]. On the contrary, no nucleic acid fragmentation was observed in negative controls represented by untreated cells. All together, these results indicate that CF induced cancer growth inhibition is occurred by the promotion of apoptosis.

**Figure 5:**
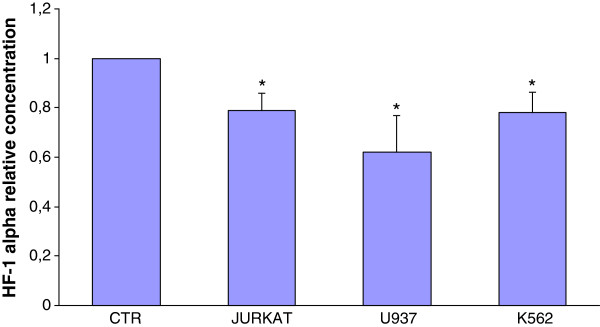
**Significant decrease of HIF-1α concentration in leukemia cells after 72 h of incubation with CF (5 μl/ml) in comparison with untreated cells (control).** Data are expressed as mean ± SD of at least three independent experiments. *p < 0.05 vs. untreated cells.

Then we wondered if apoptosis induction by CF was related to HIF-1α regulation; in fact, this transcription factor, by inhibiting the conversion of pyruvate to acetyl-CoA via the activation of pyruvate dehydrogenase kinase 1, leads to a decrease of mitochondrial oxidative phosphorylation and, consequently, to tumor cell resistance to apoptosis [35]. Our data revealed that CF treatment led to a significant reduction of HIF-1α concentration in comparison with untreated cells (Figure 5). The reduction of the transcription factor reached up to 40% in U937 cell line. Consequently, decreased levels of HIF-1α in leukemia cells treated with CF could be reasonably responsible for metabolic changes in cancer cells (from glycolysis to oxidative phosphorylation), making them susceptible to cell death, depending apoptosis on mitochondrial ATP production [11]. Based on our evidence, further studies should be conducted to confirm the activation of mitochondrial oxidative metabolism in cancer cells upon CF administration; nonetheless, in support of this hypothesis, previous observations indicated that CF administration to normal endothelial cells (HUVEC) allowed optimal O_2_ consumption by improving respiratory metabolism and mitochondrial activity [22].

**Figure 6:**
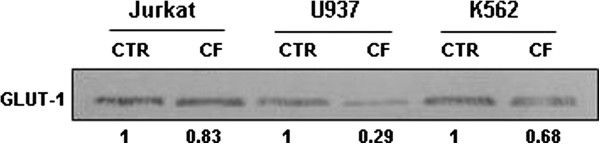
**Western Blotting analysis of GLUT-1 receptor in Jurkat, U937, and K562 leukemia cell lines after 72 h of incubation with CF (5 μl/ml) as compared to untreated cells (control).** Results are representative of three independent experiments.

Aerobic glycolysis not only provides ATP as a source of energy but also precursors and reducing equivalents for the synthesis of macromolecules [36]; therefore, glucose uptake via GLUT-1 receptor is greatly enhanced in cancer cells when compared to normal cells [9,10]. GLUT-1 is considered a legitimate target for anti-neoplastic drug development; in fact, the acquisition of the glycolytic phenotype has been shown to correlate with increased tumor aggressiveness and poor patient prognosis in several tumor types [37]. We evaluated the expression of this glucose transporter by immunoblot analysis after cancer cell incubation with CF. The densitometric analysis of the bands revealed a lower GLUT-1 expression in the three leukemia cell lines in comparison with untreated cells (Figure 6), thus indicating decreased glucose uptake in CF treated cells. The reduction of GLUT-1 expression as a consequence of CF administration was up to 70% in U937 cells.

**Figure 7:**
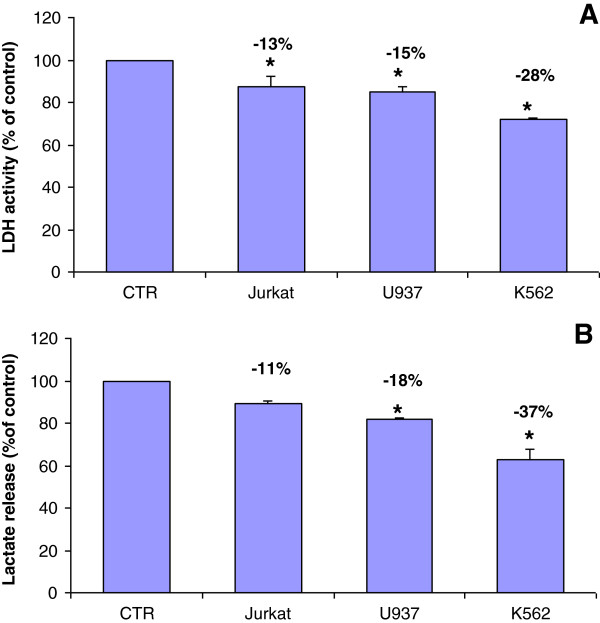
**LDH activity (A) and lactate release in the culture medium (B) in leukemia cells after 72 h of incubation with CF (5 μl/ml) in comparison with untreated cells (control).** Data are expressed as mean ± SD of at least three independent experiments. *p < 0.05 vs. untreated cells.

Other than GLUT-1 up-regulation, the activation of HIF-1 also contributes to the conversion of glucose to lactate. In fact, when stabilized, HIF-1α is directly involved in the overexpression of many glycolytic enzymes as well as LDH, the NADH-dependent enzyme that catalyzes the conversion of pyruvate to lactate [38]. Based on the observed strong LDH dependency for tumor proliferation from both in vitro and in vivo studies [39,40], inhibition of LDH may represent an alternative strategy toward the development of anti-glycolytic-based therapeutic strategies for the treatment of cancer. Noteworthy, our data revealed that CF induced a significant decrease in LDH activity after 72 hours from its administration (up to 28%) (Figure 7A). At the same time, the amount of lactate released in the extracellular environment was also reduced in CF treated cells as compared to untreated cells (up to 37%) (Figure 7B).

The reversion of the glycolytic phenotype is known to render tumor cells susceptible to apoptosis and decrease their growth rate [15-17]. In this context, our findings are in accord with recent observations indicating that the *in vitro* inhibition of tumor cell survival (T cell lymphoma) by compounds targeting tumor metabolism was accompanied by a modulation of lactate concentration in the tumor-conditioned medium, by altered expression of HIF-1α and by an alteration in the expression of apoptotic (such as caspase-3) and cell survival regulatory molecules (such as GLUT-1) [17].

Another important control point might be the glycolytic enzyme glyceraldehyde 3-phosphate dehydrogenase (GAPDH) [41]. If the oxygen supply is normal, NADH reducing equivalents that are generated by GAPDH are transported inside the mitochondria in order to reach the respiratory chain. In hypoxic conditions, the above reducing equivalents are used by LDH to convert pyruvate into lactate and no ATP can be produced into the mitochondria. This reaction is prominent in tumor cells, thus the evaluation of CF effect on GAPDH activity could also be of great interest.

Being CF a nutritional supplement that contains different active principles including deuterium sulphate, minerals, amino acids and enzymes (Everett Storey’s formula), at the moment we hypothesize that the herein described effects on leukemic cells might depend on the whole formula rather than on one or more particular components.

We also observed that the three leukemia cell lines showed different responses after CF treatment. In particular, U937 cells seemed to be the most sensitive line upon CF administration, showing the highest reduction of cell viability as well as the highest caspase-3 activation and GLUT-1 expression decrease, as compared to Jurkat and K562 cells. These findings should be probably due to the different metabolic features of the three leukemic lines; in fact, Jurkat cells are an immortalized line of T lymphocytes, while K562 and U937 cells are myelogenous leukemia lines, the first with erythroid features and the second with monocyte properties.

## Conclusions

Modulation of cell signaling, apoptotic pathways and tumor metabolism by dietary agents and nutraceutical compounds may provide new opportunities in both prevention and treatment of cancer. Herein we supply evidence for a significant antiproliferative effect of the nutritional supplement Cellfood™ on leukemia cell lines by inducing cell death through an apoptotic mechanism and by altering cell metabolism through HIF-1α and GLUT-1 regulation. Thanks to its antioxidative and proapoptotic properties, CF might be a good candidate for cancer prevention. Large-scale clinical trials will be needed to validate the usefulness of this agent either alone or in combination with the existing standard care.

## Abbreviations

CF: Cellfood™; GLUT-1: Glucose transporter 1; HIF-1α: Hypoxia inducible factor 1 alpha; LDH: Lactate dehydrogenase.

## Competing interests

The authors declare that there are no conflicts of interest.

## Authors' contributions

SC carried out the majority of the experiments and drafted the manuscript under the supervision of SBe. VC contributed to the microscopic and spectrophotometric evaluations. FP and MA carried out agarose gel electrophoresis and Western blotting. RG, BN and SBa contributed to cell culture, interpretation of data and study coordination. FC conceived the study and participated in its design and coordination. SBe performed the study design, data acquisition and analysis, and manuscript writing. All authors read and approved the final manuscript.
